# Mono-Professional Simulation-Based Obstetric Training in a Low-Resource Setting: Stepped-Wedge Cluster Randomized Trial

**DOI:** 10.2196/54911

**Published:** 2025-05-09

**Authors:** Anne A C van Tetering, Ella L de Vries, Peter Ntuyo, E R van den Heuvel, Annemarie F Fransen, M Beatrijs van der Hout-van der Jagt, Imelda Namagembe, Josaphat Byamugisha, S Guid Oei

**Affiliations:** 1 Department of Electrical Engineering, Eindhoven University of Technology, Eindhoven, The Netherlands; 2 Department of Obstetrics and Gynaecology, Amphia Ziekenhuis, Breda, The Netherlands; 3 Department of Obstetrics and Gynaecology, Máxima Medical Center, De Run 4600Veldhoven, 5504 DB, The Netherlands, 31614800853; 4 Department of Maternal Fetal Medicine, Mulago Specialised Women and Neonatal Hospital, Kampala, Uganda; 5 Dean of Mathematics & Computer Science, Eindhoven University of Technology, Eindhoven, The Netherlands; 6 Department of Biomedical Engineering, Eindhoven University of Technology, Eindhoven, The Netherlands; 7 Department of Obstetrics and Gynaecology, School of Medicine, Makerere University College of Health Sciences, Kampala, Uganda

**Keywords:** obstetric, simulation, training, low income, middle income, LMIC, simulation training, medical education, mono-professional, obstetric training, low-resource setting, low-income setting, stepped-wedge, RCT, obstetric care, pregnancy care, Africa, maternal, perinatal, outcome, hospital, train-the-trainer, facilitator, trainer, learner, mortality rate, randomized controlled trial

## Abstract

**Background:**

Emergency obstetric simulation-based training has increasingly been used to improve emergency obstetric care provision in sub-Saharan Africa. For determining the optimal methodology for effective training sessions in resource-constrained settings, it is crucial to conduct high-quality research.

**Objective:**

We aim to investigate the impact of a train-the-trainer model for providing technology-enhanced, mono-professional, simulation-based training in obstetrics in a resource-constrained setting on maternal and perinatal outcomes.

**Methods:**

A stepped-wedge cluster randomized trial was conducted from October 2014 until March 2016 at the medium- to high-risk ward at Mulago National Referral Hospital, Uganda, with an annual delivery rate of over 23,000. The intervention consisted of a train-the-trainer model in which training was cascaded down from master trainers to local facilitators (obstetric senior staff members) to learners (senior house officers). The training of senior house officers was provided to 7 fixed clusters by a computer-generated random sequential roll-out. The training comprised a 1-day (8 h), mono-professional, simulation-based training in obstetrics, and half-day repetition training sessions targeted at every 7 weeks. Both medical technical skills and teamwork skills were taught. The primary outcome comprised a combined maternal and perinatal mortality rate. Secondary outcomes comprised the maternal mortality rate, the perinatal mortality rate, the percentage of births by vacuum extraction and cesarean section, and the Weighted Adverse Outcome Score.

**Results:**

Overall, there were 17,496 births. The combined mortality rate was 9.05% (95% CI 8.37%‐9.77%) in the intervention group, and 8.73% (95% CI 8.21%‐9.28%) in the control group (odds ratio [OR] 0.98, 95% CI 0.86‐1.12; *P*=.81). No statistically significant change was found in the maternal mortality rate (OR 0.80, 95% CI 0.27‐2.32; *P*=.68) or the perinatal mortality rate (OR 0.99, 95% CI 0.87‐1.13; *P*=.87). This study did not identify any difference in the percentage of vacuum extractions, the percentage of cesarean sections, or Weighted Adverse Outcome Scores.

**Conclusions:**

This train-the-trainer model for providing technology-enhanced, mono-professional, simulation-based training in obstetrics was not able to change maternal and perinatal mortality outcomes. This study, in combination with literature, suggests that future research should consider multiprofessional team training in obstetrics involving all staff within their units.

## Introduction

### Emergency Obstetric Care in Uganda

Uganda continues to face challenges in providing safe obstetric care. Despite an increase in the rate of institutional births from 59% to 74%, the maternal mortality ratio was still high at 375 per 100,000 live births in 2017, accompanied by a neonatal mortality rate of 21 deaths per 1000 live births [1]. Key barriers for providing safe childbirth include shortcomings in the management of emergency obstetric care, delays in referral practices, and insufficient coordination among health care staff, all of which obstruct the provision of adequate emergency obstetric care [2].

### Simulation-Based Obstetric Training

To address these challenges, simulation-based training for emergency obstetric care has evolved as a promising approach in sub-Saharan Africa. Growing evidence suggests that this type of training improves health care providers’ knowledge and skills, while also leading to positive changes in their behavior [3-5]. Additionally, evidence from other studies has shown encouraging effects on patient outcomes, including reported reductions in neonatal and perinatal mortality rates, as well as potential decreases in maternal mortality and postpartum hemorrhage [6-9]. Despite these promising findings, assessments of patient outcomes remain infrequent, and the results are often inconsistent [3,4].

### Evaluating Simulation-Based Training

One limitation of current evaluations is the reliance on 1-group pretest-posttest designs, which often fail to control for external variables that may influence the results. Furthermore, significant variability exists in training length, content, and design, with programs ranging from mono-professional to multi-professional approaches. This variation makes it difficult to identify which components most effectively contribute to the success of the training. Additional challenges, such as resource constraints, difficulties in sustaining training programs, staff shortages, and high turnover rates, further hinder the implementation and long-term impact of simulation-based training in sub-Saharan Africa. To overcome these challenges, high-quality research is essential to determine the most effective methodologies for emergency obstetric simulation-based training in sub-Saharan Africa.

This study aimed to evaluate the effect of a train-the-trainer program designed to provide technology-enhanced, mono-professional, simulation-based obstetric training on patient outcomes in Uganda [10].

## Methods

### Setting

A stepped-wedge cluster randomized trial was conducted from October 2014 until March 2016 at the medium- to high-risk labor ward at Mulago National Referral Hospital in Uganda. This hospital also functions as the main teaching facility for Makerere University College of Medicine and Health Sciences. During this study’s period, over 23,000 women gave birth annually at the medium to high-risk labor ward.

### Design and Recruitment

The stepped-wedge cluster randomized trial design facilitated the phased implementation of the training program, with different clusters receiving the intervention at different periods to assess its impact on patient outcomes. This approach allowed for the measurement of the intervention’s effect both within and between clusters. Additionally, it enabled the intervention to be provided as a standard service to all participants, while being implemented in stages [11]. As training of all obstetric ward staff was not feasible due to financial and logistical challenges, senior house officers (SHOs) were chosen as the target group for the training program due to their coordinating role in providing emergency obstetric care.

**Figure 1.**
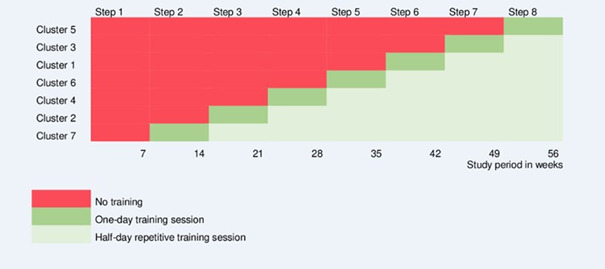
Stepped-wedge cluster randomized design with 7 clusters and 8 steps.

In October 2014, a total of 7 fixed clusters of SHOs were recruited to receive the training. All participants provided written informed consent before this study began. A computer-generated random sequential roll-out of the training program was conducted to determine the order in which the different clusters would receive the intervention (Figure 1). Examination and holiday periods were excluded from the schedule, as fixed clusters could not be maintained in the SHOs’ work schedules during these times.

### Train-the-Trainer Model

The training program was conducted using a train-the-trainer model, in which training was cascaded down from master trainers to local facilitators, and then to the learners, who were the SHOs. In this model, the master trainers, who were obstetricians from a high-resource setting, had been previously certified as simulation-based trainers by institutions such as EuSim or the Center for Medical Simulation. These master trainers provided a 4-day train-the-trainer program to 14 local facilitators. The facilitators, all gynecologists, were selected based on their clinical and teaching experience by the head of the department. The program included both lectures and practical teaching sessions using simulation-based obstetric scenarios. The train-the-trainer course concluded with an assessment session. During this session, the local facilitators trained intern doctors using a draft SHO training program. Afterward, the master trainers provided feedback to the facilitators. Based on this, 12 of the facilitators were certified as simulation trainers. The SHO training program was then adjusted based on feedback from both trainers and trainees. Subsequently, the local facilitators delivered the training to fixed clusters of 6 to 9 SHOs, comprising first-, second-, and third-year SHOs. A 1-day annual refresher training was offered to all local facilitators. The local facilitators were compensated for lost clinical income by being paid for their participation in the training sessions.

### Course Content

Course content was developed by Medsim, a medical simulation center in the Netherlands, in cooperation with senior staff members of Mulago National Referral Hospital. The SHO training program included a 1-day (8 h) mono-professional, simulation-based sessions, followed by half-day refresher sessions every 7 weeks. These refresher sessions started after the switch from the control to the intervention group. Each training session was provided by 2 local facilitators. Scenarios were based on the main local causes of maternal and perinatal mortality and tailored to local clinical protocols and availability of medical equipment. This led to the creation of 2 different scenarios for postpartum hemorrhage, a scenario for eclampsia, a scenario involving fetal distress with a ventouse delivery, and a breech delivery scenario. Both medical-technical and teamwork skills were included in the training, with the difficulty level increasing throughout the day. Every SHO participated in at least 2 scenarios during the 1-day training, while having an observer role in the nonparticipating scenarios. During the repetition training sessions, a single clinical scenario was executed and repeated until skills were mastered.

### Data Collection and Outcomes

The primary outcome of this study was the combined maternal and perinatal mortality rate, expressed as a percentage of maternal and perinatal deaths per total number of births. Perinatal deaths were defined as stillbirths and deaths occurring within the first week of life in the special care unit. Data about each delivery and maternal and perinatal outcomes were prospectively registered using the maternity register and transcribed without identification of the subjects. Data about maternal deaths in the high dependency unit, and neonatal deaths in the special care unit were obtained from registration books in these units. These data were matched to and merged with data from the maternity register of the medium to high-risk ward into 1 final electronic database.

Secondary outcomes comprised the maternal mortality rate (maternal deaths per 100,000 births), the perinatal mortality rate (perinatal deaths per 1000 births), percentage of births by vacuum extraction, percentage of births by cesarean section, and the Weighted Adverse Outcome Score (WAOS). The WAOS was defined as the total weighted score of each adverse outcome divided by the total number of births [12]. Four out of 10 index measures (maternal death [750 points], intrapartum or perinatal death [400 points], uterine rupture [100 points], Apgar score less than 7 after 5 minutes [25 points]) were available for registration and assessment. Finally, the maternal mortality ratio (maternal mortality per 100,000 live births), and the perinatal mortality ratio (perinatal mortality per 1000 live births) were calculated for the control and intervention group. As data were analyzed on the cluster level, the authors could not identify individual participants’ results.

To provide a comprehensive understanding of the training’s effectiveness, additional secondary outcomes were included and published separately, such as the evaluation of the instructional design, participants’ reactions (corresponding to Kirkpatrick level 1), and the effects on knowledge, teamwork, and medical-technical skills (corresponding to Kirkpatrick level 2) [13].

### Sample Size Calculation

The power calculation was conducted following the methods described by Hussey et al [14] and Woertman et al [10,15,16]. Initially, the sample size for a standard randomized clinical trial was calculated. To show a 20% reduction in combined maternal and perinatal mortality with an α of .05 and a power of 80%, a total of 6398 births would be required for a simple randomized clinical trial design. The design effect was then determined, assuming an intracluster correlation of 0.05, a cluster size of 3343 births per year, and 7 clusters in total. Accounting for the design effect, 2367 births per measurement period would be required. To meet this target, each measurement period would need to last at least 5 weeks. However, for logistical feasibility, the duration of each step was set at 7 weeks, resulting in a total study duration of 56 weeks. Statistical significance was defined as a 2-sided *P* value of <.05.

### Statistical Analysis

Patient baseline characteristics were summarized with medians and IQRs for continuous variables and with counts (percentages) for categorical variables. A generalized linear mixed-effects model was used for the estimation of an intervention effect. Here, the outcome is the binary event on the individual (whether a birth was or was not complicated by maternal mortality, perinatal mortality, or both), and a logit link function was used to model the probability of the event. In the logit scale, the cluster indicator served as a random effect on the intercept, and the period of the stepped wedge was treated as a fixed effect, as well as the intervention effect. The effect of the intervention was reported as an odds ratio, and the performance per treatment group was reported as a percentage or as an incidence rate ratio with 95% CI.

### Ethical Considerations

Ethical approval was obtained from the Mulago Research and Ethics Committee (Protocol MREC: 674), and the Uganda National Council for Science and Technology (UNCST, SS 3927). Written informed consent to participants in this study was obtained at the beginning of the first training day, and all participants had the ability to opt out during this study’s period. No compensation was provided for the participants. All data were anonymized.

## Results

**Figure 2.**
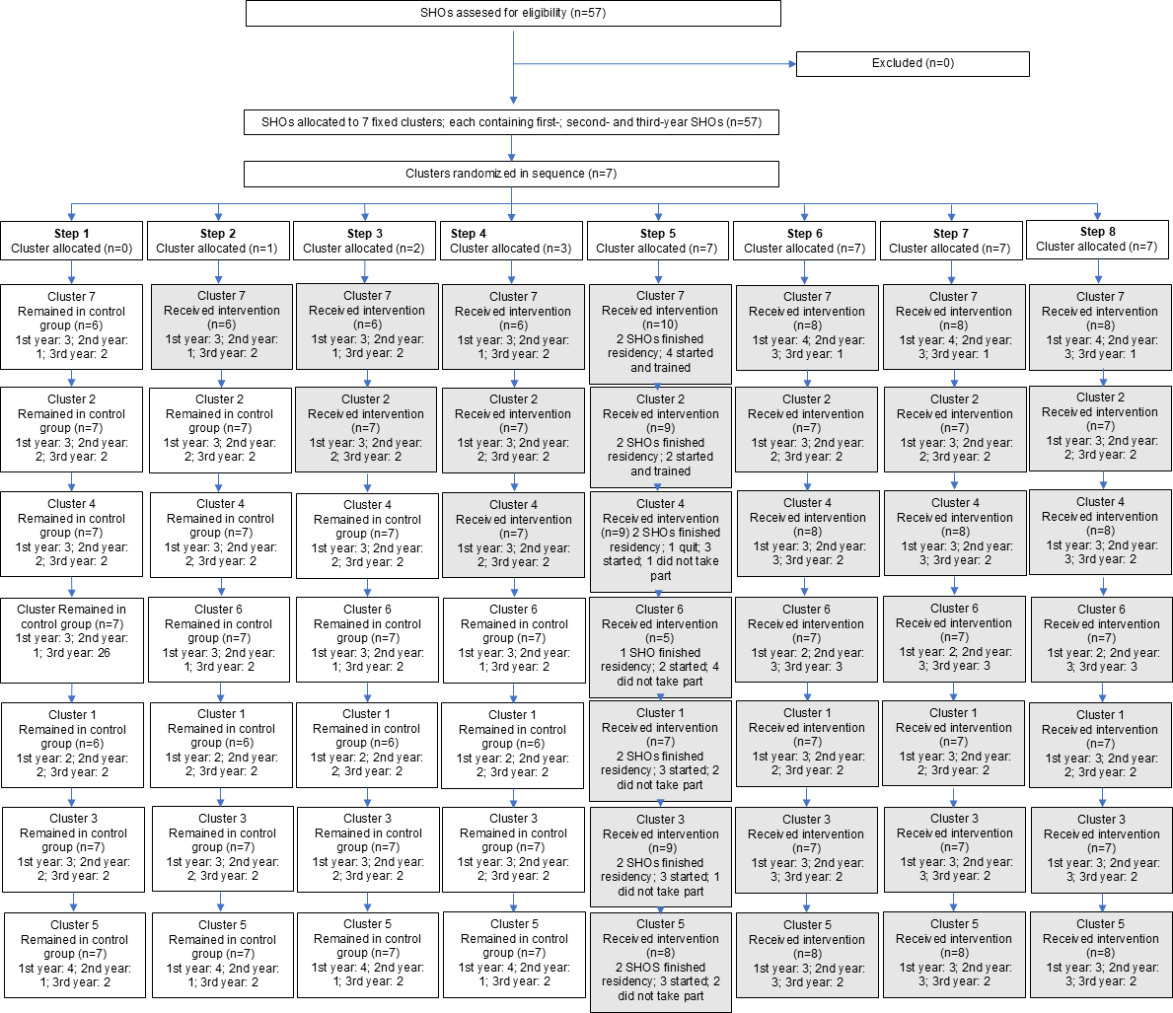
Flowchart of stepped-wedge cluster randomized design. SHO: senior house officer.

From October 2014 until March 2016, a total of 57 SHOs were randomized into 7 clusters. The first 3 included clusters received the main training on schedule according to protocol. Afterward, when SHOs heard about the training experience from their peers, they requested to expedite the training schedule, rather than waiting for the allocated time slot. Therefore, these 4 remaining clusters were trained within the same week and they simultaneously switched from the control group to the intervention group during this week (Figure 2). After the deviation from the stepped-wedge design in the timing of the intervention, no amendment was needed by the ethical committee, as the intervention itself had not changed.

Baseline characteristics are shown in Table 1. Overall, there were 17,496 births. There were fewer female and more male neonates in the intervention group compared to the control group. The results on maternal and perinatal mortality rates, mode of delivery, and the WAOS are shown in Table 2. No differences were found between the intervention and the control group in the combined maternal and perinatal mortality rate, the maternal mortality rate, and the perinatal mortality rate. Results on the percentage of vacuum extractions, the percentage of cesarean sections, and the WAOS did not show any difference between the intervention and the control group. The maternal mortality ratio in the control and intervention group was, respectively, 160.4 (95% CI 94.9‐266.6) and 116.9 (95% CI 51.2‐252.4). The perinatal mortality ratio in the control and intervention group was 94.4 (95% CI 88.8‐100.4) and 98.7 (95% CI 91.3‐106.6).

**Table 1.** Baseline characteristics.

Characteristics			Total	Control group	Intervention group	*P* value
Maternal characteristics						
	Age (years), median (IQR)		24 (21-28)	24 (21-28)	24 (21-29)	.11^a^
	Parity, n					.71^b^
		Primiparous	6760	4219	2541	
		Multiparous	9594	5960	3634	
	Gestation, n (%)					.18^b^
		Preterm (<37 weeks)	1183 (8.2)	753 (8.5)	430 (7.8)	
		Full-term (>37 weeks)	13,215 (91.8)	8149 (91.5)	5066 (92.2)	
	Pregnancy, n (%)					.09^b^
		Singleton	16,166 (92.4)	10,099 (92.5)	6067 (92.2)	
		Twin	1299 (7.4)	795 (7.3)	504 (7.7)	
		Triplet	30 (0.2)	24 (0.2)	6 (0.1)	
Neonatal characteristics						
	Gender, n (%)					.002^b^
		Female	8381 (48.4)	5328 (49.3)	3053 (46.9)	
		Male	8926 (51.6)	5471 (50.7)	3455 (53.1)	
	Birth weight (kg), median (IQR)		3.1 (2.7-3.4)	3.1 (2.6-3.4)	3.1 (2.7-3.4)	.70^a^

a Wilcoxon rank sum test.

b Pearson chi-square test.

**Table 2.** Study results.

	Total	Before intervention	After intervention	Odds ratio	*P* value
Combined mortality rate, % (95% CI)	8.9 (8.4‐9.3)	8.7 (8.2‐9.3)	9.1 (8.4-9.8)	0.98 (0.86 to 1.12)	.81
Maternal mortality rate, event rate per 100,000 births (95% CI)	131.5 (85.3‐200.6)	146.5 (86.7‐243.6)	106.4 (46.6‐229.7)	0.80 (0.27 to 2.32)	.68
Perinatal mortality rate, event rate per 1000 births (95% CI)	87.6 (83.5‐91.9)	86.3 (81.1‐91.7)	89.8 (83.1‐97.1)	0.99 (0.87 to 1.13)	.87
Births by vacuum extraction, % (95% CI)	2.3 (2.1‐2.6)	2.43 (2.2‐2.8)	2.15 (1.8‐2.5)	1.00 (0.76 to 1.33)	.99
Births by cesarean section, % (95% CI)	26.6 (25.9‐27.2)	26 (25.2‐26.8)	27.5 (26.4‐28.6)	1.06 (0.94 to 1.2)	.33
Weighted Adverse Outcome Score (WAOS), median score (IQR)	39.6 (0‐282.6)	39.1 (0‐280.8)	40.5 (0‐285.5)	−0.59 (−5.22 to 4.04)^a^	.80

a Difference.

## Discussion

### Principal Results

This train-the-trainer model for providing technology-enhanced, mono-professional, simulation-based obstetric training to SHOs did not result in changes to maternal and perinatal mortality outcomes. The training program also had no impact on the number of instrumental births, the number of cesareans, or the WAOS.

### Strengths and Limitations

A strength of this study was the use of a randomized stepped-wedge trial design, enabling the training for all SHOs in one of the busiest labor wards worldwide. Unlike 1 group pretest-posttest designs commonly used in prior research on obstetric simulation-based training, this approach minimized bias from natural changes in health care outcomes because it could eliminate systematic period effects. Another strength was the use of the train-the-trainer model. Research has shown that training delivered by local trainers results in greater improvements in knowledge and skill acquisition [17]. Furthermore, simulation-based learning is likely to be more effective when tailored to the local context and culture [18]. A third strength of the evaluated training program was the inclusion of teamwork skills. Teamwork skills are increasingly recognized as a critical factor in reducing preventable, substandard care and are viewed as an essential competence for hospital teams [19]. These skills had not been part of previous SHO training programs at Mulago National Referral Hospital.

Limitations of our study should also be considered. First, the intended stepped-wedge design was altered during the study, as clusters requested earlier training. Although this deviation interfered with the planned study design, it was deemed unethical to withhold training further. The change in design did not affect the statistical analysis because the mean features of the stepped wedge design were maintained. Other studies have highlighted ethical concerns with the stepped-wedge design, including justifying the delayed rollout of the intervention to the control group, which is inherent to this design [20,21]. A potential solution in the future could involve using different hospitals as clusters. This approach would also address the challenge of maintaining fixed clusters of individuals during working hours, which was one of the difficulties we encountered. Interaction between trained and nontrained SHOs during shifts in examination and holiday periods may have introduced bias into the results, but our analysis was chosen conservatively, making sure that such biases would affect the treatment effect negatively. Another challenge in the work schedules of the fixed clusters was the repetition of sessions, which led to some sessions not being scheduled according to the protocol. Establishing fixed clusters of multidisciplinary obstetric team members within 1 hospital is anticipated to be even more challenging. Including different hospitals in the design could eliminate these issues and allow all health care providers involved in maternity and neonatal care at a single hospital to be trained within a short period. However, it may be difficult to include hospitals with comparable levels of care and delivery volumes, which should be accounted for in the intracluster correlation coefficient and the statistical analysis.

### Comparison With Prior Work

The results of our study on maternal and perinatal outcomes do not align with those of recent studies on simulation-based emergency obstetric training in sub-Saharan Africa. Since the start of this study, 6 studies reported improvements in maternal outcomes, mainly related to postpartum hemorrhage and mortality [22-27], and 7 studies showed improvements in neonatal or perinatal outcomes after simulation-based obstetric training [25,28-33]. One of these studies showed that initial improvements declined over time [29].

When comparing our simulation-based training program to others that were effective, we want to highlight the mono-professional nature of our program. While previous research showed that simulation-based team leader training improved teamwork and communication during clinical resuscitations, our study found that training only SHOs as the leaders during obstetric emergencies did not improve patient outcomes. This aligns with the findings of Siassakos et al [34], who noted that units showing improvements had trained nearly 100% of their staff and implemented training programs within their own units. Additionally, all but 2 of the previously described effective studies were multi-professional training programs [22-31]. An exception to justify a mono-professional training program can be when the focus is on a specific technical task performed by a single health care provider, such as repairing an episiotomy. In such cases, the focus on a specific task allows for deliberate practice, where the trainee improves the task through immediate feedback, problem-solving, evaluation, and repeated performance. However, when the task involves teamwork, the training approach should shift toward a multi-professional model. In conclusion, our results, alongside the literature, suggest that future research should consider multi-professional team training in obstetrics, involving all staff within their units.

Another difference between our training program and others is that ours was a stand-alone program, while simulation-based training as part of an integrated package may be more effective in improving health outcomes [4,8,28]. Integrated packages often include equipment, maternal death reviews, health information system improvements, modified protocols, supportive supervision, mobile mentoring, and peer-assisted learning. Although we included a train-the-trainer model, restructured local protocols, and created posters with flowcharts for obstetric emergencies, the intensity and, ultimately, the training frequency of our intervention may not have been sufficient to impact maternal and neonatal outcomes.

Another notable variance between evaluated simulation-based training programs is the location of training. Our study used an off-site medical simulation center, while on-site training may be more beneficial, as it reaches more staff and generates more suggestions for organizational changes. Sorensen et al [35] found no significant differences in knowledge, patient safety attitude, motivation, or stress between on-site and off-site training, but the on-site group suggested more organizational changes. In low-resource settings, these changes may be more valuable, although on-site training could be disrupted by clinical situations. Further research comparing on-site versus off-site training in low-resource settings would be valuable.

A further difference in previous studies on simulation-based obstetric training is the definition of mortality ratios. Some studies, including the mortality ratios in the introduction, used maternal mortality per live birth, while others used maternal mortality per number of births [8,22,26,36,37], making comparisons difficult. Additionally, the World Health Organization defines maternal and perinatal mortality ratios with different denominators, complicating statistical analyses of a combined mortality ratio. As a result, we analyzed the combined mortality rate and reported the maternal and perinatal mortality ratios separately. Moreover, the ratios are based on live births, so improving perinatal care can also affect maternal mortality outcomes. A standardized approach to mortality ratios could improve the comparability of future training programs.

A final note should be made regarding improvements in data administration. It took considerable time to manually collect, verify, and process all the data. In some cases, determining the time and cause of death was challenging, potentially leading to the inclusion of more macerated babies and higher perinatal mortality rates. This study’s setting in a national underresourced referral hospital may also explain the high perinatal mortality rate. Options to address some data challenges include the development of digital data registration systems and active surveillance of data. While these solutions require initial investments of both time and money, they offer significant potential benefits in terms of efficiency and accuracy. Additionally, digital data registration and continuous data monitoring can be enhanced by the use of dashboards, which provide clinicians with an overview of current practices and can help identify deviations from targets early on [38]. This approach not only benefits the evaluation of obstetric simulation-based training but can also inform ongoing training sessions, contributing to continuous learning and improvements in obstetric training.

Given the complexity of simulation-based obstetric training implementation and evaluation in low-resource settings, future studies should consider conducting implementation and action research. This approach would be valuable for identifying barriers to effective implementation, refining training programs, and ensuring that improvements are successfully integrated into the local health care system.

### Conclusions

This train-the-trainer model for providing technology-enhanced, mono-professional, simulation-based training in obstetrics to SHOs did not change maternal and perinatal mortality outcomes in a national referral hospital in a low-resource setting. This study, along with existing literature, suggests that future research should consider conducting and evaluating multi-professional team training in obstetrics, involving all staff within their units.

## Supplementary material

10.2196/54911 Checklist 1 CONSORT checklist. CONSORT: Consolidated Standards of Reporting Trials.

## Abbreviations

OR: odds ratio
SHO: senior house officer
WAOS: Weighted Adverse Outcome Score
